# Sustained posterior negativity (SPN) elicited by brief (20 ms) symmetrical stimuli

**DOI:** 10.3389/fpsyg.2025.1657531

**Published:** 2026-02-13

**Authors:** Giulio Contemori, Marianna Musa, Deniz Demirkapi, Martina Passaggi, Carolina Maria Oletto, Luca Battaglini, Marco Bertamini

**Affiliations:** Department of General Psychology, University of Padova, Padova, Italy

**Keywords:** sustained posterior negativity (SPN), event-related potentials, symmetry, visual regularity, visual perception

## Abstract

**Introduction:**

Mirror symmetry is detected rapidly, yet it remains unresolved whether its canonical neural signature—the Sustained Posterior Negativity (SPN)—requires extended viewing. We therefore asked whether symmetry-selective activity can be generated under highly constrained temporal conditions that limit both prolonged processing and premotor preparation.

**Methods:**

We presented centrally displayed, irregular octagons (symmetric or asymmetric) for 20 ms while preventing premotor preparation via a delayed, trial-wise randomized response mapping. EEG was recorded with a 64-channel montage and preprocessed using a fully scripted, automated pipeline; ERPs were computed from correct trials only. Symmetry-related activity was first assessed with a mass-univariate, cluster-based permutation analysis, and then quantified in a confirmatory analysis within a canonical posterior region of interest. A fixed-cohort trial-subsampling analysis was used to examine how per-condition epoch count affects SPN estimation.

**Results:**

The cluster-based permutation analysis revealed a mid-latency posterior negativity with the occipito-parietal topography characteristic of the SPN, and the canonical posterior region-of-interest analysis converged on the same effect. Behaviorally, participants discriminated symmetry reliably with a modest liberal bias. Fixed-cohort trial subsampling showed that increasing per-condition epochs enhanced statistical detectability without altering amplitude, indicating precision gains rather than changes in the underlying response. No reliable condition differences were observed in earlier sensory components, and there was no compelling hemispheric lateralization.

**Discussion:**

These results demonstrate that brief visual evidence is sufficient to initiate symmetry-selective activity that unfolds over the established SPN time course in the absence of sustained viewing or response preparation. This pattern supports a hybrid account in which a rapid feedforward sweep flags global regularity while recurrent interactions sustain and consolidate the representation, thereby tightening temporal constraints on the neural mechanisms that extract structural order from transient input.

## Introduction

Humans and other animals exhibit remarkable efficiency in perceiving symmetry, even under conditions of minimal visual input. Foundational psychophysical work (Barlow and Reeves, 1979; Julesz, 1971) demonstrated that human observers can distinguish symmetrical from random patterns after very brief exposures. Using forced-choice paradigms, Tyler (1995) identified detection thresholds as low as approximately 40 milliseconds for static mirror symmetry and around 80 milliseconds for dynamic alternations, with participants performing significantly above chance levels. More recently, Sharman and Gheorghiu (2018) corroborated these findings by showing that symmetry can be detected in displays presented for just 30–50 milliseconds, reinforcing the view that symmetry perception recruits a rapid and automatic visual mechanism.

However, although symmetry can be detected “at a glance,” increased exposure duration yields systematic gains in perceptual sensitivity. Signal detection metrics, such as d′, continue to improve with longer viewing times. Wenderoth (1996) reported that while symmetry discrimination performance exceeded chance at 60 milliseconds, it improved further at 100–200 milliseconds, typically reaching 75–80% accuracy, contingent on stimulus type. This performance suggests perceptual sensitivity in the range of d′ ≈ 0.8–1.3. Similarly, Cohen and Zaidi (2013) found that while observers could detect the orientation of a symmetry axis as early as 28 milliseconds, robust sensitivity (d′ ≈ 1.0) emerged only after 60–80 milliseconds. Studies that explicitly chart d′ as a function of exposure time (e.g., Sharman and Gheorghiu, 2018; Tyler, 1995) consistently show a steep increase in symmetry perceptibility beginning near 40 milliseconds, followed by a plateau around 100 milliseconds. Such patterns support a functional dissociation: while early detection may rely on a rapid feedforward sweep, extended viewing enables refinement and discrimination—particularly when the input is noisy or ambiguous (van der Helm and Treder, 2009; Wagemans, 1995).

This temporal profile invites questions regarding the underlying mechanisms. While some accounts posit that symmetry is detected during the initial feedforward pass through the visual system, without feedback or top-down influence (Keefe et al., 2018), a growing body of work favors a hybrid model. Specifically, symmetry perception—especially under degraded or ambiguous conditions—relies on spatial integration and feedback loops involving higher-order visual and attentional areas (Machilsen et al., 2009; Makin et al., 2024). Under such suboptimal conditions, symmetry detection becomes increasingly dependent on attention and expectation (Treder, 2010), and does not exhibit the automaticity typically associated with perceptual “pop-out.” In visual search tasks, symmetry detection is strongly modulated by features such as axis orientation, symmetry type, and global configuration regularity (Moreau et al., 2025; Olivers and Van Der Helm, 1998; Van Zoest et al., 2006). In reaction time studies, closure facilitates perception of symmetry (Bertamini, 2010; Bertamini et al., 1997). Computational studies further reinforce this view, showing that feedforward convolutional networks struggle to generalize symmetry, whereas recurrent architectures better approximate human performance (Sundaram et al., 2022).

These behavioral and computational findings align with neurophysiological evidence that links symmetry perception to a distinct electrophysiological signature: the Sustained Posterior Negativity (SPN). The SPN is a negative-going waveform elicited over posterior electrodes by symmetrical as compared to asymmetrical patterns, typically emerging around 220–250 milliseconds post-stimulus and persisting for several hundred milliseconds (Höfel and Jacobsen, 2007; Norcia et al., 2002). Most studies define the SPN within a 250–600 ms time window (Makin et al., 2022; Makin et al., 2024), although in some cases it has been shown to extend up to 1,000 ms (Makin et al., 2012; Norcia et al., 2002). Recent analyses suggest a biphasic structure, with an early phase (~250–450 ms) maximal over posterior-occipital sites and a later phase (~450–600 ms) that shifts anteriorly (Dering et al., 2024; Wright et al., 2017). In addition, figure–ground organization can modulate its timing: symmetry carried by the ground region delays SPN onset, plausibly because object-formation processes precede and interfere with symmetry extraction (Karakashevska et al., 2021). Taken together, these observations support a functional differentiation whereby the early SPN reflects perceptual encoding of regularity, whereas the later phase indexes post-perceptual operations such as categorization or working-memory maintenance.

Crucially, the timing and duration of the SPN argue against a purely brief feedforward sweep. Its amplitude is systematically modulated by attention, task demands, and signal quality, consistent with feedback-supported consolidation of global structure. Directing attention to symmetry enhances the SPN, whereas adverse visual noise diminishes it (Alp et al., 2018; Palumbo et al., 2015), and degradations of perceptual clarity further attenuate the response (Dering et al., 2024). Task constraints can also alter SPN lateralization and overall strength (Tyson-Carr et al., 2025). Moreover, causal interventions implicate extrastriate generators: transcranial magnetic stimulation over the lateral occipital complex (LOC) disrupts symmetry discrimination without perturbing early sensory potentials, linking the SPN to high-level ventral visual areas rather than to early feedforward encoding alone (Bona et al., 2014; Cattaneo et al., 2011). Together, these findings converge on the interpretation that the SPN arises from recurrent, feedback-driven consolidation of global structure within visual cortex.

At the same time, the SPN is both automatic and resilient (Zamboni et al., 2024). It appears robustly across a range of conditions, even when symmetry is not task-relevant (Höfel and Jacobsen, 2007; Jacobsen and Höfel, 2003; Makin et al., 2012, 2024). Divided-attention paradigms continue to elicit SPNs, and adding a demanding working-memory task does not abolish the effect (Derpsch et al., 2021, 2024). Nevertheless, symmetry perception—and the SPN—remains sensitive to how readily the visual system can establish an appropriate reference for matching corresponding parts, which in turn depends on the availability and predictability of candidate axes.

In this respect, the number and predictability of symmetry axes exert systematic effects on behavior (Palmer and Hemenway, 1978; Treder, 2010; Wenderoth, 1997), with direct consequences for neural responses when axis selection is uncertain. Multiple equivalent axes (for example, vertical and horizontal) facilitate detection relative to a single axis, presumably because at least one axis aligns with the observer’s current template (Palmer and Hemenway, 1978; Wenderoth, 1997). Consistently, priming or cueing the likely axis improves performance by pre-aligning the coordinate system before part-matching is evaluated (Chen and Tyler, 2010; Wenderoth, 1996; Yamauchi et al., 2006), and the SPN itself grows across rapid sequences of different reflection exemplars, indicating cumulative facilitation under conditions that stabilize axis selection (Makin et al., 2021).

Object-centered reference frames further shape these dynamics: observers exploit an intrinsic frame defined by a shape’s principal (often longest) axis to support rapid, global comparisons in search and recognition (Boutsen and Marendaz, 2001). When reflectional symmetry coexists with pronounced elongation, the elongation axis typically dominates as the operative frame, yielding a “winner-take-all” outcome in which the most salient axis prevails (He and Ogmen, 2024; Sekuler and Swimmer, 2000). This dominance is a mixed blessing: when the dominant object-based frame coincides with the symmetry axis, organization is efficient; when it is misaligned, the system must re-frame the object, slowing decisions and increasing errors (Treder, 2010). Electrophysiologically, the SPN mirrors this bottleneck: it becomes larger when spatial attention or an informative cue aligns with the correct axis, and weaker when axis orientation is uncertain (Makin et al., 2021; Tyson-Carr et al., 2022). In sum, these regularities motivate a two-stage account in which an initial axis-selection step establishes the coordinate frame for evaluating mirror correspondences, upon which sustained symmetry-selective activity builds (Palmer and Hemenway, 1978; Treder, 2010).

### Present study rationale: brief symmetry exposure

**Figure 1:**
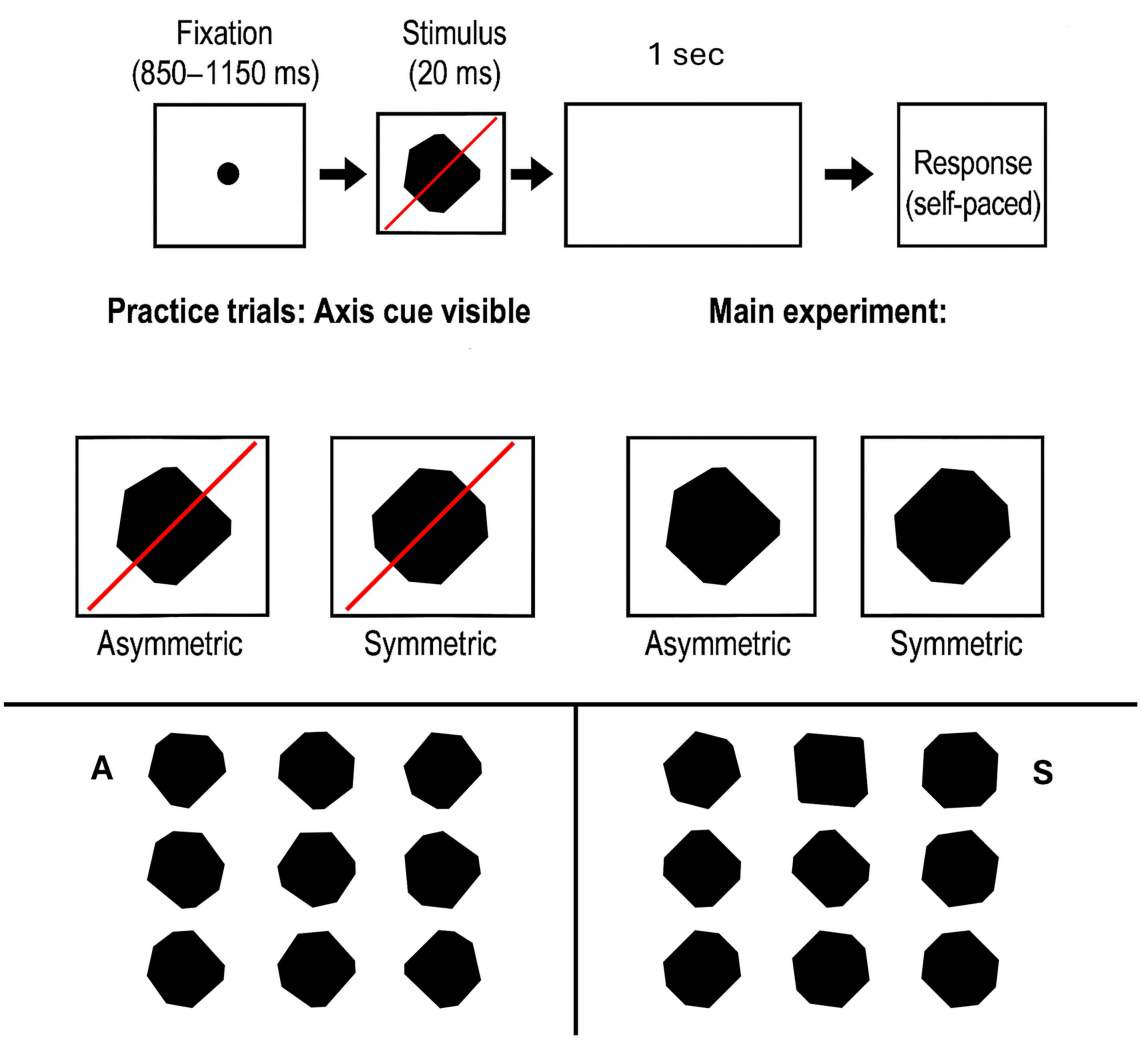
Each trial began with a variable fixation period (850–1,150 ms), followed by a brief presentation of an octagonal shape (20 ms). A blank screen was then displayed for 1,000 ms, after which participants provided an unspeeded symmetry judgment. In practice trials only, a red line indicated one axis of potential symmetry to facilitate task understanding; this cue was removed for the main experiment. All symmetric octagons possessed two orthogonal axes of mirror symmetry, oriented at 45° and 135°. Critically, this rotation ensured that the internal reference frame of the stimulus (specifically, the major axis of elongation) could align with either one of these symmetry axes, making that axis more salient than the other, but it never overlapped with the cardinal axes of the display. The delayed response was used to minimize EEG contamination from motor-related activity and to allow for the isolation of post-stimulus neural responses.

The present study was designed to test whether the visual system can generate the Sustained Posterior Negativity (SPN)—a reliable neural marker of symmetry perception—even when stimuli are presented too briefly to support prolonged processing or top-down modulation. To achieve this, we asked participants to discriminate symmetrical and asymmetrical octagonal shapes and applied two key constraints to the task. First, stimuli were presented for only 20 ms, an exposure far shorter than the typical onset of the SPN (~250 ms; Bertamini and Makin, 2014; Makin et al., 2022; Norcia et al., 2002). Second, the symmetrical stimuli were constructed in such a way that they were frequently elongated along one axis (see Method section for details). This elongation both distorted and defined an object-centered reference frame whose orientation and salience varied randomly across trials, rendering the stimuli less predictable than completely regular octagons. When the object’s principal elongation axis overlapped with one of the symmetry axes, that axis tended to dominate perceptual organization, rendering it more salient. As illustrated in Figure 1, the resulting stimuli preserved mirror symmetry but varied systematically in contour and in the relative dominance of competing axes. To minimize reliance on the external reference frame of the display, the two symmetry axes were oriented obliquely (45° and 135°), preventing alignment with either the cardinal axes or the screen’s diagonals (Treder, 2010; Wenderoth, 1996). Although feedback processes cannot be entirely ruled out, the short presentation strongly restricts the temporal window available for recurrent activity (Lamme and Roelfsema, 2000).

Importantly, any neural activity occurring after target onset—including visual transients or the initiation of motor preparation—can interfere with or obscure SPN. To eliminate such contamination, participants were not only instructed to delay their response, but were also prevented from preparing any motor action, as the correct response mapping (left vs. right key) was revealed only after a one-second interval. This ensured that the EEG signal during the SPN window remained uncontaminated by lateralized motor potentials and could be attributed exclusively to perceptual integration mechanisms.

Together, these methodological features define a constrained environment in which top-down modulation and prolonged visual input are minimized. This offers an opportunity to isolate the minimal conditions required for SPN generation. If a robust and fully expressed SPN emerges despite the brief and unpredictable stimuli, this would indicate that feedforward input is sufficient for initiating and sustaining symmetry-selective neural activity. In contrast, if no SPN is observed, it would support the view that feedback and temporal integration are necessary for constructing global representations of regularity (Alp et al., 2018; Machilsen et al., 2009).

Critically, if the SPN is present but substantially attenuated, this would suggest that feedforward processing is sufficient to trigger SPN onset, but that feedback mechanisms are necessary for full consolidation and for achieving the typical magnitude of the response. This would imply a hybrid dependency in which initial symmetry detection operates automatically via early visual pathways, but full engagement of symmetry-sensitive networks—especially those associated with sustained activity in ventral visual areas—relies on reentrant and top-down amplification. Such an outcome would also align with the known temporal profile of the SPN: even though the stimulus is visible for only 20 ms, the SPN typically begins around 250 ms and extends well beyond, making it likely that recurrent processing contributes to its full expression.

This is the first study to attempt eliciting the SPN under such brief exposure. Although symmetry-related effects have occasionally been reported in earlier ERP components (e.g., P1 or N1, Dering et al., 2024; Makin et al., 2012), these lack the temporal and functional specificity of the SPN, which reflects a sustained perceptual response to global regularity. Nevertheless, an early feedforward contribution cannot be excluded *a priori*. Accordingly, we adopted an agnostic approach and tested explicitly for symmetry-related activity at any latency and scalp distribution. To this end, we used a nonparametric, data-driven cluster-based permutation analysis, which enables unbiased detection of spatiotemporal clusters in the EEG signal where responses to symmetrical and asymmetrical stimuli diverge, providing a statistically rigorous and assumption-light means of identifying the presence, onset, and topography of symmetry-related neural activity.

Finally, as a confirmatory analysis, we computed the SPN using a canonical literature-derived posterior ROI (O1, O2, Oz, PO3, PO4, PO7, PO8) and the conventional 250–600 ms window. This spatiotemporal definition is widely adopted in the SPN literature and summarized in recent reviews and catalogues (Bertamini et al., 2018; Dering et al., 2024; Derpsch et al., 2024; Makin et al., 2022; Wright et al., 2015).

## Method

### Participants

24 participants (14 female; mean age = 24.83 years, SD = 4.81) were recruited from the University of Padova. All participants reported normal or corrected-to-normal vision and no history of neurological or psychiatric disorders. Each provided written informed consent. The study protocol was approved by the university of Padova ethics committee (Comitato etico della ricerca psicologica—area 17), protocol number 588-a (17/05/2024). Data collection formed part of a broader research initiative investigating metacontrast masking.

### Stimuli and experimental design

The experimental sessions took place in a quiet, dimly lit room. Participants were seated 57 cm from a calibrated PHILIPS100 LCD monitor (60 cm width, 1920 × 1,080 resolution, 100 Hz refresh rate), with head position stabilized via a chinrest to maintain a fixed viewing geometry. Stimuli were generated in PsychoPy3 (version 2021.2.3; Peirce et al., 2019).

Octagonal stimuli for both the symmetric (S) and asymmetric (A) conditions were generated dynamically with a parametric algorithm. The base template was a regular octagon inscribed in a circle of radius 0.75° of visual angle (1.5° diameter). Each of the eight vertices was independently rotated about the origin by an angular jitter uniformly sampled from −20° to +20°, keeping radial distance fixed but producing irregular, non-equidistant contours. This ensured that no perfectly regular octagon was ever displayed.

The two conditions differed only in how these vertices were constrained. In the asymmetric condition, all eight vertices were independent, yielding stimuli with no mirror- or rotational-symmetry aside from rare accidental configurations. In the symmetric condition, two anchor vertices were random and the remaining six were generated by successive reflections across the vertical and horizontal axes. After construction, the stimulus was rotated by either 45° or 135°, so the symmetry axes (originally vertical and horizontal in object coordinates) appeared at 45° and 135° in display coordinates. Importantly, these orientations did not coincide with the monitor diagonals (e.g., ~29°/151° on a 16:9 display), preventing the screen’s reference frame from serving as a cue. Because the same base geometry and radial extent were used in both conditions, average global size, perimeter, and enclosed area were matched, minimizing luminance and contrast-energy confounds.

Both S and A stimuli were elongated (anisotropy). In A stimuli, elongation simply established a principal (object-centered) axis without introducing mirror structure. In S stimuli, elongation sets the object reference frame to align with one of the two oblique symmetry axes (45° or 135°), and thus to be misaligned with the other. This trial-to-trial variability in which axis captured the reference frame prevented the adoption of fixed strategies or canonical templates. The task therefore required trial-by-trial global shape extraction to detect symmetry under minimal visual input and variable geometry.

Each trial followed a fixed temporal sequence. A central black fixation dot (≈ 0.2° visual angle) appeared on a uniform mid-gray background for a variable duration (850–1,150 ms, mean = 1,000 ms), after which a single filled black octagon was presented centrally for exactly 20 ms (two frames at 100 Hz). Participants were instructed that a geometric shape would briefly appear at fixation on each trial and were asked to judge whether it was symmetric or asymmetric.

A critical design feature of the present paradigm was the trial-by-trial randomization of the mapping between symmetry judgments and motor responses. Specifically, participants were informed that the labels “symmetric” and “asymmetric” would be variably assigned to either the left or right arrow key on each trial. To eliminate anticipatory motor preparation and ensure that the SPN reflects perceptual rather than premotor activity, the response mapping cue (e.g., “LEFT = symmetric; RIGHT = asymmetric” or its reverse) was presented only after stimulus offset—a protocol consistent with best practices in symmetry-related ERP research (Makin et al., 2022). This post-stimulus cueing effectively decouples lateralized readiness potentials (LRPs) from the post-perceptual time window of interest, thereby preserving the interpretive specificity of the SPN as an index of symmetry detection.

After reading the instructions, participants pressed the spacebar to begin and then completed a practice block designed to familiarize them with the brief, irregular stimuli and with the delayed, trial-wise response mapping. The practice block replicated the main task in all respects: the same fixation jitter (850–1,150 ms), the same 20 ms stimulus duration, a 1 s blank interval after stimulus offset, the same post-offset display of the key mapping, and the same centrally presented octagons. Only two brief instructional elements were added during practice. First, immediate accuracy feedback was presented for 1 s after each response (“Correct” in green; “Incorrect” in red). Second, during the 20 ms stimulus, a thin red oblique guideline was momentarily superimposed along one potential axis of symmetry; on each practice trial its orientation was 45° or 135°, selected pseudorandomly. Because the guideline orientation and the shape’s geometry (including any elongation that defined an object-centered reference axis) varied from trial to trial, the guideline always coincided with one of the two possible symmetry axes but only sometimes aligned with the object’s intrinsic reference frame.

The practice block comprised 80 trials. Because most participants were naïve to EEG, this block was essential to ensure stable task performance and proper recording behavior (e.g., minimizing blinks and muscle tension). While a slightly shorter practice might have sufficed, this dose remains within the same order of magnitude as familiarization phases commonly used in SPN studies, where practice typically consists of a few dozen trials (sometimes repeatable to criterion) and includes trial-level feedback to stabilize performance before the no-feedback main task (e.g., Karakashevska et al., 2024; Rampone et al., 2022).

In the main experiment, participants completed 120 pseudorandomized trials. No feedback or axis cues were presented; between trials the screen contained only a fixation point. No backward masking followed the 20 ms stimulus, ensuring that EEG responses during the SPN window were driven solely by the brief shape onset.

### EEG recording and preprocessing

Electrophysiological data were recorded with a 64-channel actiCAP arranged according to the extended 10–20 international system. Preprocessing was fully scripted and executed in EEGLAB v2024.2 (Delorme et al., 2011) running in MATLAB R2023a. Continuous data were resampled to 256 Hz and then band-pass filtered from 0.5 to 40 Hz using zero-phase Hamming-windowed sinc FIR filters to minimize phase distortion. To attenuate mains-related line noise, CleanLine was applied in multi-taper mode with adaptive sinusoid regression at 50 and 100 Hz (Mullen, 2012).

Automated continuous-data cleaning proceeded with the clean_rawdata/ASR framework (Kothe et al., 2019) using the following a-priori criteria applied uniformly across participants: FlatlineCriterion = 10 s, ChannelCriterion = 0.80 (correlation-based channel outlier detection), LineNoiseCriterion = 4.5, High-pass range for ASR calibration = 0.25–0.75 Hz, BurstCriterion = 30, WindowCriterion = 0.35 with WindowCriterionTolerances = [−Inf, 10], and BurstRejection = ‘off’. Channels flagged at this stage were removed for decomposition and later restored by interpolation.

Independent Component Analysis (ICA) was performed with the Picard algorithm, after PCA reduction to the number of retained channels to ensure full rank. Components were classified with ICLabel (v1.3) and automatically rejected when their class probabilities exceeded the following thresholds: Muscle ≥ 0.95, Eye ≥ 0.80, Heart ≥ 0.80, Line noise ≥ 0.95, Channel noise ≥ 0.95, and Other ≥ 0.95; no component was rejected based on “Brain” probability (Pion-Tonachini et al., 2019).

After ICA-based artifact removal, previously removed channels were re-interpolated to the original 64-channel montage using spherical splines. Data were then re-referenced to the common average computed over scalp EEG channels only.

Continuous EEG was epoched from −500 to +1,000 ms relative to stimulus onset for asymmetric (“A”) and symmetric (“S”) trials, and a baseline correction was applied using the −200 to 0 ms interval. In keeping with best practice for stimulus-locked ERPs, only correct trials were retained for ERP averaging: behavioral accuracy codes were aligned to epochs, and incorrect trials were removed automatically after epoching. Remaining epochs then underwent a fully automated rejection pass based on scalp channels only (EOG excluded): trials were rejected if they exceeded ±120 μV within −200 to 600 ms, exhibited a linear trend with slope > 75 μV and R^2^ > 0.50, or showed kurtosis > 7 SD at either the local- or global-channel level.

Across the 24 participants processed with this automated pipeline, the mean number of channels removed by the continuous cleaning stage and subsequently re-interpolated was 5.25 per participant (range: 3–9). The number of independent components rejected by ICLabel-guided automation ranged from 1 to 8, with a mean of 4.08 components per participant. Behavioral exclusion of incorrect responses removed on average 24.04 trials per participant from the initial 120, after which the automated epoch-quality screen flagged and removed a further 2.46 trials on average (≈ 2.5% of available epochs; range: 0–6). The final number of retained epochs per participant was therefore 93.5 on average (SD = 13.52; range: 57–111). Condition-wise, participants contributed on average 44.88 asymmetric trials (range: 28–55) and 48.63 symmetric trials (range: 25–57), which were adequate for stable ERP estimation in the predefined occipito-parietal regions of interest, as demonstrated by a reliability analysis of SPN amplitudes as a function of per-participant epoch count reported in the Results section. All preprocessing steps, parameters, and decision rules were applied uniformly by the scripted workflow, ensuring full reproducibility and eliminating rater-dependent variability from the pipeline.

### Cluster-based permutation testing

To identify spatiotemporal clusters reflecting sustained symmetry-related responses, we performed a non-parametric, within-subject cluster-based permutation test. For each participant, trials were first averaged within condition to obtain subject-level time courses at every electrode. Condition differences were then evaluated with paired (two-tailed) mass-univariate tests computed at each electrode–timepoint.

Spatiotemporal clusters were defined as contiguous sets of electrode–time samples whose univariate tests exceeded a cluster-forming threshold of *p* < 0.05. Spatial adjacency was based on inter-electrode distances, treating sensors separated by < 35 mm as neighbors, yielding on average ~3.3 neighbors per electrode; as expected for peripheral sites, TP9 and TP10 had no valid neighbors. This neighborhood structure balances anatomical specificity with the expected spatial spread of scalp potentials in high-density EEG (Maris and Oostenveld, 2007). Family-wise error was controlled at the cluster level using a Monte Carlo permutation procedure (5,000 random label-swaps). On each iteration, condition labels were exchanged within participants to simulate the null hypothesis while preserving the within-subject design. For every permuted dataset, the maximum cluster mass (sum of test statistics over each spatiotemporally contiguous cluster) was recorded to form the empirical null distribution of maximal effects. Observed clusters were deemed significant if their mass fell in the extreme 5% of this distribution (two-sided, cluster-level *α* = 0.05).

This approach produces binary spatiotemporal masks marking samples that belong to statistically reliable clusters and supports conservative, data-driven inference about the timing and scalp distribution of the sustained posterior negativity without imposing *a priori* constraints on latency or topography.

### Quantification of the sustained posterior negativity (SPN)

We quantified symmetry-related activity as the SPN, defined as the mean ERP amplitude difference between symmetric and asymmetric trials (S − A) computed over posterior electrodes within a specified time window. In the data-driven analysis, both the electrode set and the latency window were taken directly from the significant spatiotemporal cluster identified by the permutation test (see above). For each participant and condition, we first averaged the ERP within this window at each electrode in the cluster, yielding one mean per electrode per condition. We then computed electrode-wise difference scores (S − A) and averaged these across the cluster electrodes to obtain a single SPN value per participant.

For completeness and comparability with prior work, we also computed a canonical SPN using a literature-based posterior ROI (O1, O2, Oz, PO3, PO4, PO7, PO8) and the conventional 250–600 ms window. The same steps were applied: per-condition averaging within the canonical window at each ROI electrode, electrode-wise S − A differencing, and averaging of these differences across ROI electrodes. Both the cluster-defined and canonical SPN estimates were carried forward, with the former serving as the primary, data-driven definition and the latter as a robustness check.

## Results

According to a one-tailed binomial test at α = 0.05, at least 69 correct responses out of 120 trials (accuracy ≥ 0.575) were required to exceed chance performance. One participant fell below this threshold and was excluded from all subsequent analyses, yielding a final sample of 23 participants. The remaining dataset comprised 2,760 valid trials (23 × 120).

We modeled trial-level accuracy with a binomial generalized linear mixed-effects model (GLMM) using a logit link and a random intercept for participant (intercept-only fixed structure). A Wald χ^2^ test on the fixed intercept indicated accuracy was well above chance, *χ*^2^(1) = 160.03, *p* < 0.001. The logit intercept estimate was 1.554 (SE = 0.123), *z* = 12.65, *p* = 0.001, corresponding to an estimated group-level accuracy of 0.825, 95% CI [0.788, 0.858]. These results confirm high performance at the group level.

**Figure 2:**
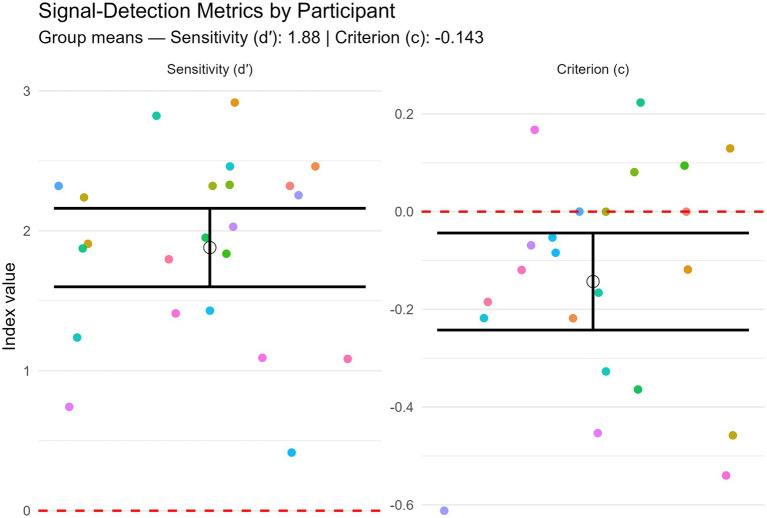
Signal-detection metrics by participant. Left: sensitivity (d′). Right: decision criterion (c). Each dot is one participant; colors are consistent across panels. The white disk and black error bar show the intercept-only (grand-mean) estimate with its 95% CI. The red dashed line marks 0 (no sensitivity for d′; unbiased criterion for c). Negative c indicates a liberal bias; positive c indicates a conservative bias.

Signal-detection analyses separated perceptual sensitivity (d′) from decision criterion (c). Mean sensitivity was d′ = 1.88 ± 0.65 SD, indicating robust discrimination of symmetry from asymmetry, while the mean criterion was c = −0.14 ± 0.23 SD, reflecting a modest liberal bias. Both metrics differed significantly from zero (*t* (22) = 13.90, *p* < 0.001; *t* (22) = −2.99, *p* = 0.007, respectively). Figure 2 illustrates individual and group-level distributions for these two indices: each participant is represented by a single dot (dot colors are consistent across panels and figures), and the white disks with black error bars indicate the grand-mean estimates with their 95% confidence intervals. The dashed red lines mark theoretical neutrality (no sensitivity for d′, unbiased criterion for c). The figure thus summarizes participants’ consistent ability to detect symmetry alongside a small tendency to favor the “symmetric” response under uncertainty.

### Cluster-based evidence for the SPN

**Figure 3:**
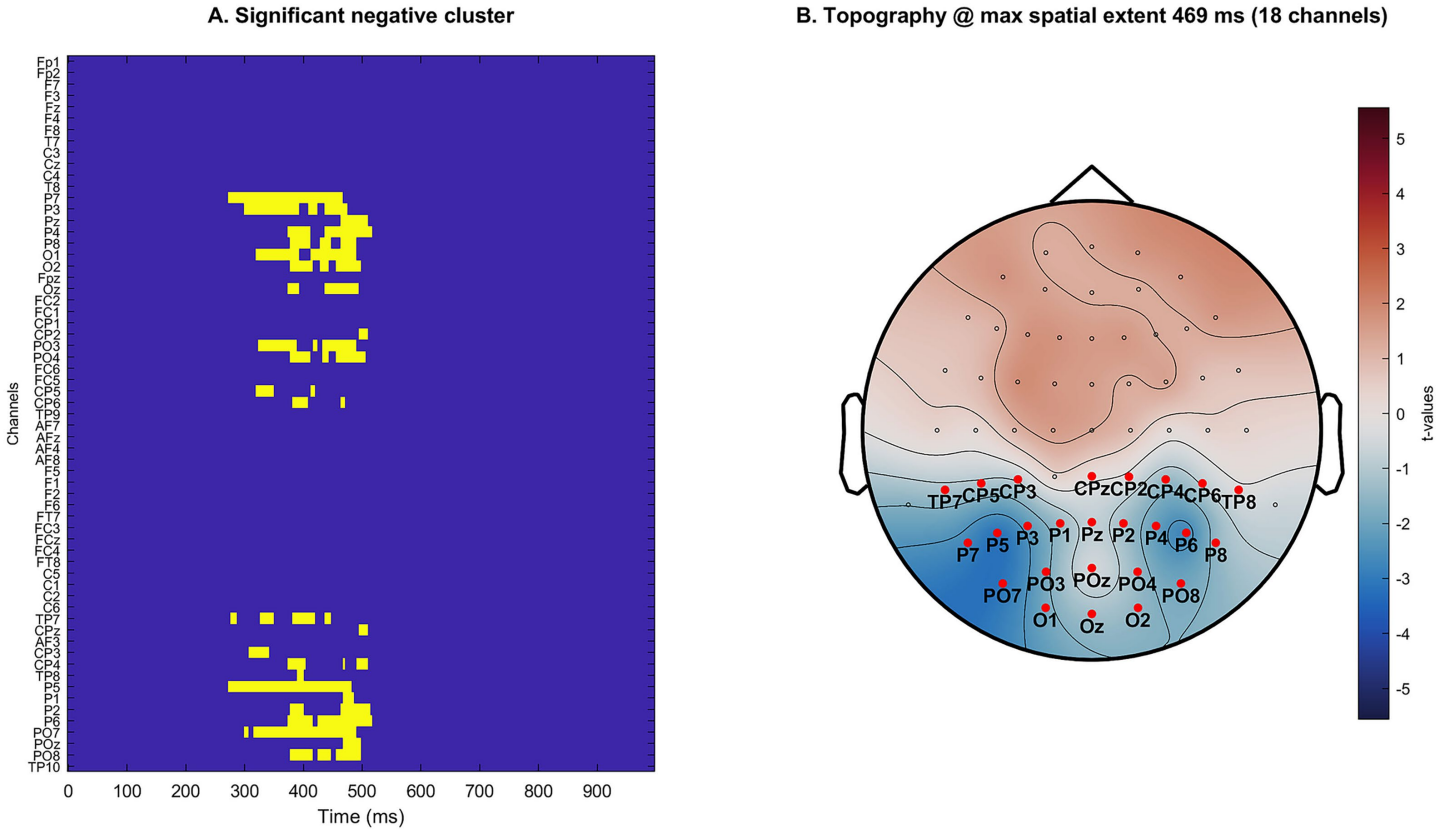
Spatiotemporal cluster of ERP differences (Symmetric − Asymmetric). Panel **(A)** shows the binary mask over channels (*y*-axis) and time (*x*-axis, 0–1,000 ms) indicating samples that contributed to the significant negative cluster (S < A). Panel **(B)** depicts the corresponding scalp topography, averaged across the cluster window (309–508 ms; cluster-corrected *p* = 0.002). Topographies display interpolated sensor-level *t*-values (Symmetric minus Asymmetric), with red markers labeling the electrodes belonging to the cluster at the time of maximal spatial extent (383 ms; 14 channels).

The non-parametric, trial-level cluster permutation analysis revealed a single statistically significant negative spatiotemporal cluster, consistent with an SPN pattern in which responses to symmetric stimuli were more negative than to asymmetric stimuli. The cluster extended from 309 to 508 ms post-stimulus (duration ≈ 199 ms), encompassed 25 posterior occipito-parietal electrodes (P7, P3, Pz, P4, P8, O1, O2, Oz, CP2, PO3, PO4, CP5, CP6, TP7, CPz, CP3, CP4, TP8, P5, P1, P2, P6, PO7, POz, PO8), and reached a maximal spatial extent of 14 contributing electrodes at 383 ms. The cluster-level probability, corrected for multiple comparisons, was *p* = 0.002. No positive cluster (Symmetric > Asymmetric) survived correction (see Figure 3).

### ERP evidence for the SPN in the cluster-based spatiotemporal ROI

**Figure 4:**
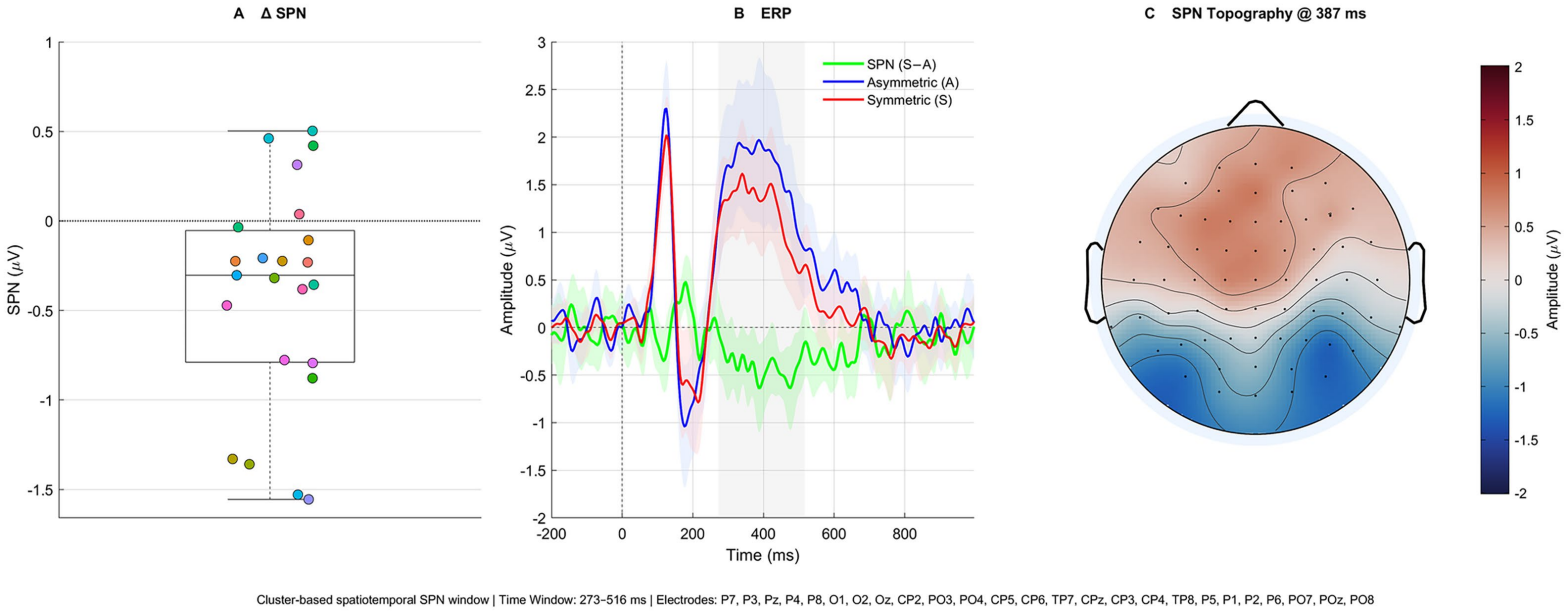
Cluster-based SPN summary (273–516 ms). **(A)** Cluster-based SPN. Box-and-jitter of each participant’s window-averaged SPN (Symmetric − Asymmetric) within the cluster-based ROI (25 electrodes: P7, P3, Pz, P4, P8, O1, O2, Oz, CP2, PO3, PO4, CP5, CP6, TP7, CPz, CP3, CP4, TP8, P5, P1, P2, P6, PO7, POz, PO8). The dashed line marks 0 μV. **(B)** ERP (ROI). Grand-average ROI waveforms for Symmetric (red), Asymmetric (blue), and their difference SPN = S − A (green). Shaded bands are 95% confidence intervals across participants (participant is the sampling unit after averaging within participant × condition). The gray band marks the cluster-defined analysis window (309–508 ms; cluster permutation, *p* = 0.002). **(C)** SPN topography at peak. Scalp map of the grand-average SPN at the latency of maximal negativity within the window (≈387 ms, −0.636 μV), showing a posterior distribution with strongest negativity over occipital–parietal sites (color bar at right = μV). Sample size: *N* = 23.

To estimate SPN amplitude at the individual level, we computed each participant’s mean ERP difference (Symmetric − Asymmetric) within the spatiotemporal extent of the negative cluster window (309–508 ms), which was defined by the cluster-based permutation test rather than *a priori* assumptions (FieldTrip cluster *p* = 0.002). As shown in Figure 4A, the mean SPN across participants (*N* = 23) was −0.446 μV (median = −0.397 μV). The interquartile range spanned −0.669 to −0.036 μV, indicating that most individuals showed a clear negativity with relatively few null or reversed effects. Maximal negativity within the window occurred at ≈387 ms (≈ − 0.636 μV). A one-tailed one-sample t-test confirmed that the SPN was less than zero, *t* (22) = −3.390, *p* = 0.001; the one-sided 95% CI was (−∞, −0.173] μV and the two-sided 95% CI was [−0.719, −0.173] μV. The corresponding within-subject effect size was d_z_ = −0.71 (medium to large).

To characterize the scalp distribution, we located the time of maximal negativity within the same window by averaging the SPN difference wave across participants and electrodes; the peak occurred at ≈387 ms. The resulting topography (Figure 4C) shows a posterior distribution with strongest negativity over occipital–parietal sites. For reference, Figure 4B displays the grand-average ROI waveforms for Symmetric (red), Asymmetric (blue), and the SPN difference (green) with 95% confidence bands.

### Hemispheric dominance of the SPN (cluster-based)

We asked whether the SPN was stronger over one hemisphere by computing two related lateralization indices (LI) within the cluster-defined window (309–508 ms) and using the same left- and right-hemisphere electrode sets.

Condition-based LI. For each participant and for each condition (Symmetric, Asymmetric), we averaged ERP amplitudes across the cluster electrodes and time points. We then computed the right–minus–left difference within each condition and combined the two conditions into a single, normalized index by dividing that difference by the sum of the absolute left and right means. This index reflects any hemispheric bias present in the overall waveforms, whether or not it is specifically tied to symmetry.

SPN-based LI. For each participant we first formed the SPN on each hemisphere (SPN_L = Symmetric minus Asymmetric on the left; SPN_R = Symmetric minus Asymmetric on the right). We then took the right–minus–left difference of these SPNs and normalized it by the sum of their absolute values. This index isolates lateralization of the symmetry effect itself, ignoring activity common to both conditions.

The SPN-based indices were near zero [*M* = 0.127, SD = 0.689, 95% CI (−0.171, 0.425), *t* (22) = 0.89, *p* = 0.385, d_z_ = 0.18]. A Bayesian one-sample test also favored the null (BF10 = 0.31; roughly 3:1 evidence for no lateralization). The condition-based index led to the same conclusion (*t* (45) = −0.21, *p* = 0.832, BF10 = 0.16).

The sustained posterior negativity elicited by 20-ms symmetric stimuli is essentially bilateral: the SPN does not show a reliable left–right asymmetry, and the raw ERPs likewise do not reveal a systematic hemispheric bias.

### Relationship between SPN and SDT metrics (d′ and c)

An important question is whether participants who exhibit a stronger neural response to symmetry (as indexed by the SPN) are also those who perform better in the behavioral task or adopt a more conservative or liberal decision strategy. To address this, we computed correlations between each participant’s SPN amplitude and their signal-detection metrics: sensitivity (d′) and decision criterion (c). SPN amplitude was defined as the mean difference between Symmetric and Asymmetric trials (S − A), averaged across the data-driven posterior NEG cluster (25 electrodes) and the cluster-defined time window (309–508 ms), which captures the sustained posterior negativity elicited by symmetry.

For perceptual sensitivity (d′), the Pearson correlation with SPN amplitude was very small (*r* = 0.062) and not statistically significant, with a 95% confidence interval (CI) that ranged from a moderately negative to a moderately positive association (−0.359 to 0.463). This interval includes zero, indicating that we cannot rule out the possibility of no association at all. The corresponding Bayesian correlation analysis produced a Bayes Factor of BF10 = 0.46, meaning the observed data were about 2.2 times more likely under the null hypothesis of no correlation (BF01 ≈ 2.2). The 95% Highest Density Interval (HDI) for the population correlation *ρ* was [−0.320, 0.403], again including zero and suggesting substantial uncertainty about the true relationship.

For the decision criterion (c), which reflects response bias, the correlation was slightly larger but still non-significant [*r* = 0.160, 95% CI (−0.270, 0.536), *p* = 0.467]. Participants who showed a stronger (i.e., more negative) SPN did not consistently adopt a more conservative or liberal response tendency. The Bayesian analysis yielded BF10 = 0.56 (BF01 ≈ 1.8), which provides only anecdotal evidence favoring the null hypothesis. The 95% HDI for *ρ* was [−0.249, 0.465], indicating that correlations in either direction remain plausible given the present sample size.

**Figure 5:**
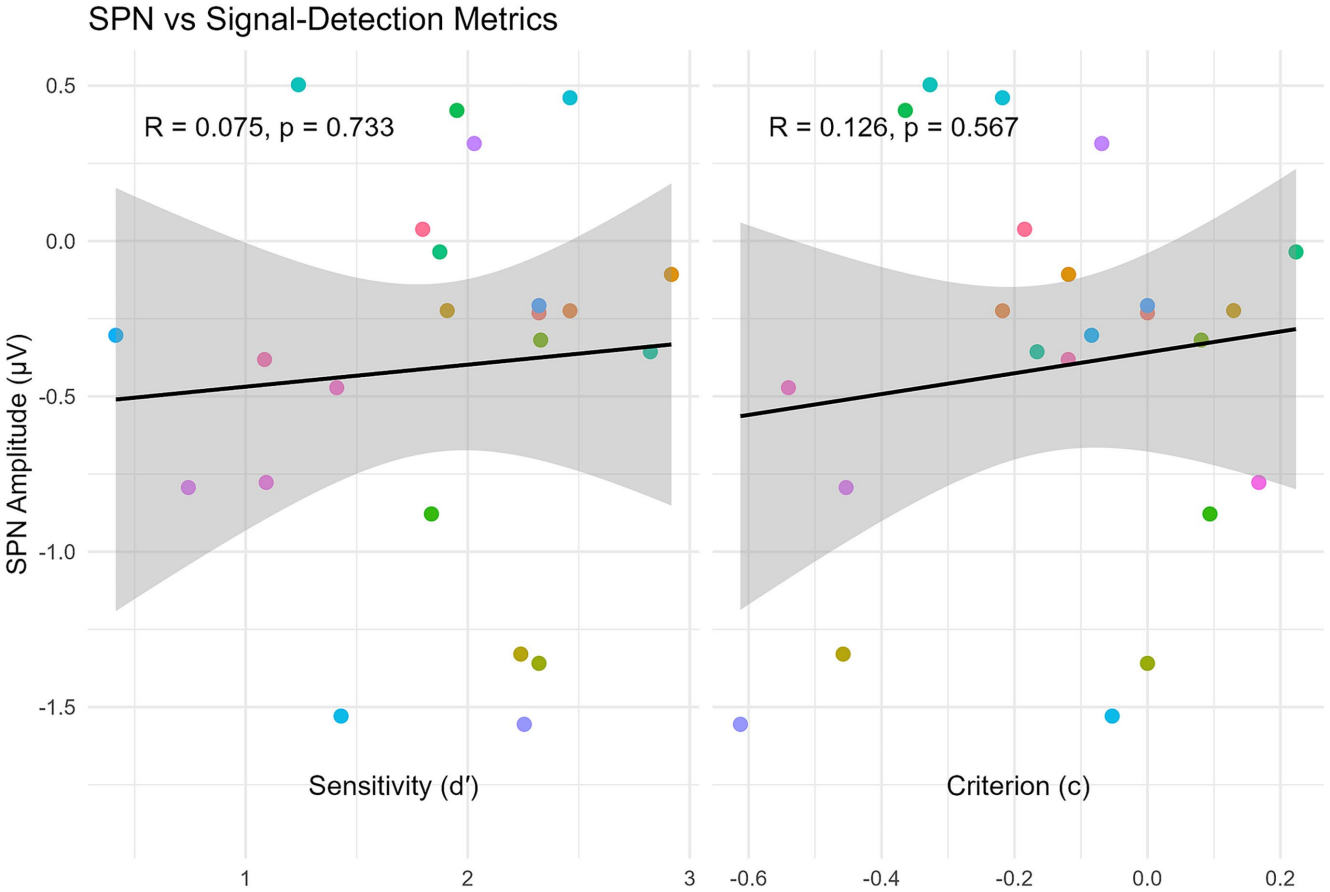
Relationship between SPN and signal-detection metrics. Each point is one participant (colors consistent across panels). SPN values are mean amplitudes (μV) computed as Symmetric minus Asymmetric and averaged over the NEG posterior cluster (P7, P3, Pz, P4, P8, O1, O2, Oz, CP2, PO3, PO4, CP5, CP6, TP7, CPz, CP3, CP4, TP8, P5, P1, P2, P6, PO7, POz, PO8) within 309–508 ms. Left: SPN versus sensitivity (d′). Right: SPN versus decision criterion (c). Black lines show least-squares fits with 95% confidence bands. More negative SPN reflects a larger symmetry-related negativity; higher d′ indicates better discrimination; negative c reflects a more liberal response bias.

In this dataset, the neural index of symmetry processing (SPN) appears largely independent of both discrimination performance and decision bias. The convergence of frequentist non-significance, confidence intervals that include zero, and Bayesian evidence favoring the null suggests that any genuine brain–behavior association—if present—is at most small to moderate. With *N* = 23 the achieved power to detect a medium correlation (*ρ* = 0.30) is modest (≈ 0.28), and the breadth of both confidence and credibility intervals counsels caution against treating the absence of an effect as definitive. Future studies should therefore employ larger samples to narrow these intervals and yield a more precise estimate of any putative association (see Figure 5).

### ERP evidence for the SPN in the canonical posterior ROI

Within the posterior ROI (O1, O2, Oz, PO3, PO4, PO7, PO8; 250–600 ms), the SPN was reliably negative. Across participants (post-exclusion *N* = 23), the mean SPN was −0.507 μV (median = −0.221 μV), with an interquartile range from −0.919 to 0.154 μV—indicating that most individuals showed a clear negativity, with few null or reversed effects. A one-tailed one-sample t-test confirmed that the SPN was less than zero, *t* (22) = −2.83, *p* = 0.005, yielding a one-sided 95% CI of (−∞, −0.199] μV [two-sided 95% CI: (−0.879, −0.135) μV]. The corresponding within-subject effect size was d_z_ = −0.59, in the medium range.

To determine the minimum number of epochs per condition needed for a reliable canonical SPN, we performed a fixed-cohort subsampling analysis on epoch-level ROI means (post-exclusion *N* = 23). We evaluated five candidate counts per condition, *m* ∈ {5, 10, 15, 20, 25}. For each *m*, we ran 1,000 permutations. Within each permutation—and within each participant—we sampled without replacement *m* symmetric and *m* asymmetric epochs, computed the participant’s SPN as mean(S) − mean(A), and tested the across-participant mean against zero with a one-tailed one-sample t-test (H₁: SPN < 0). “Reliability” was defined as the proportion of permutations with *p* < 0.05; 95% confidence intervals were exact Clopper–Pearson intervals. In parallel, for each *m* we summarized the mean SPN across permutations and its 2.5–97.5% permutation band.

Subsampling was balanced and used a fixed-cohort: every included participant contributed the same number of epochs per condition. Accordingly, the largest testable *m* is capped by the participant with the fewest balanced usable epochs (here, 25). In the present dataset, available counts were typically much higher (per-participant medians ≈ 45 for A and 51 for S; minima 28 and 25). The analysis is therefore conservative: it asks how many trials per condition are minimally sufficient for reliable detection in this cohort while preventing a few high-trial participants from disproportionately driving the result. Reliability increased monotonically with m: 30.5% (5), 45.9% (10), 64.0% (15), 79.4% (20), and 89.1% [25; exact 95% CI ≈ (0.87, 0.91)]. At m = 25, the SPN was significant in ≈89% of permutations. Across permutations, the mean SPN remained stably negative (≈ − 0.52 to −0.53 μV), indicating that improved reliability with larger m reflects reduced sampling variability rather than changes in effect magnitude.

## Discussion

Brief visual stimuli primarily evoke early, transient ERP components—most notably P1 (~100 ms) and N1 (~150 ms)—which index the initial feedforward analysis of low-level features in striate and early extrastriate cortex (Clark and Hillyard, 1996; Di Russo et al., 2003). By contrast, the sustained posterior negativity (SPN) emerges later, beginning around 220–250 ms and extending for several hundred milliseconds, and is widely interpreted as an index of the integration of local elements into coherent global structure, including symmetry (Makin et al., 2012; Norcia et al., 2002). Against this backdrop, our study asked whether a fully formed SPN can be elicited by a single 20 ms presentation. The overarching aim was to test whether symmetry-selective neural activity can arise with minimal sustained stimulation and reduced influence of predictive template allocation. A second aim was to probe whether between-participant variation in SPN magnitude bears on behavioral performance.

Behavioral performance confirmed that symmetry was accessible even under deliberately stringent conditions. Sensitivity exceeded chance, whereas the decision criterion exhibited a modest liberal shift. This configuration accords with the well-documented propensity to “see” symmetry when evidence is weak—a bias plausibly rooted in the ecological prevalence of bilateral structure and thus adaptive as a perceptual heuristic (Freyd and Tversky, 1984). The task was intentionally demanding because asymmetric shapes were matched to symmetric ones in overall appearance; had asymmetries been more salient, accuracy would have approached ceiling. As anticipated, most errors were false alarms on asymmetric trials, consistent with reports that perfect symmetry is seldom missed while subtle departures from regularity are sometimes mistakenly accepted as symmetric. Moreover, response bias in symmetry judgments is known to vary with stimulus properties—shifting toward conservatism for luminance-defined patterns and toward liberalism for isoluminant configurations (Martinovic et al., 2018).

Crucially, this behavioral profile aligns with the dissociation between early visual responses and the sustained symmetry signal observed in prior EEG work. Makin et al. (2020) showed that symmetric “hits” elicited a robust SPN that scaled with regularity, whereas symmetric “misses” preserved typical P1/N1 but lacked a sustained SPN, indicating a failure to consolidate a symmetrical Gestalt despite intact early sensory processing. False alarms produced an SPN that was detectable yet clearly weaker than that for true symmetric hits, reinforcing the interpretation that the SPN indexes veridical symmetry organization rather than generic decision tendencies. In the present study, this consideration guided the electrophysiological analysis: ERP averages were computed on correct trials only, in line with SPN conventions that target the neural signature of successful symmetry perception and thereby minimize contamination from response bias (Bertamini et al., 2019; Makin et al., 2022).

The EEG analyses converged on a sustained, posterior negativity that differentiates symmetric from asymmetric patterns in the expected latency range. We first adopted a nonparametric, cluster-based permutation test to search, without *a priori* spatial or temporal constraints, for reliable differences over the full electrode-by-time volume. This analysis identified a negative posterior cluster whose onset followed the early sensory complex and whose time course overlapped the canonical SPN window. The scalp distribution was occipito-parietal and matched the literature’s characterization of the SPN topography (Bertamini et al., 2019; Makin et al., 2022). No reliable differences were detected in earlier post-stimulus intervals, or in different electrodes. Thus, the first statistically supported divergence between conditions aligns with the onset and distribution of the SPN, rather than reflecting modulations of P1 or N1. This outcome is what would be expected if global-form integration mechanisms are engaged once the initial feedforward sweep has registered sufficient structure to support symmetry extraction.

We then tested whether this data-driven effect would be replicated under a conventional literature-derived montage and window. Re-measuring in the canonical posterior ROI over 250–600 ms (O1, O2, Oz, PO3, PO4, PO7, PO8) again yielded a reliable sustained negativity (mean SPN = −0.51 μV; d_z_ ≈ −0.59). For context, the Liverpool SPN Catalogue is a curated open resource that aggregates grand-average SPNs together with their underlying participant-level datasets and rich metadata (e.g., task relevance, stimulus type, electrode sets, analysis windows), currently comprising 249 grand-averages from 6,674 datasets across 40 projects (Makin et al., 2022). Our amplitude sits toward the lower end of that distribution—only about a third of catalogue grand-averages are ≤ 0.5 μV—and task-relevant SPNs are typically larger. A simple mechanistic account follows: the SPN is present even with extremely brief stimulation, consistent with feedforward processing being sufficient to trigger its onset; yet its attenuated magnitude suggests that recurrent/feedback processes are needed to fully consolidate regularity and reach the larger amplitudes more often observed when symmetry is salient or task-relevant.

We next considered reliability and precision. Participants contributed an average of 93 usable epochs (≈ 45 artifact-free per condition), aligning with common SPN practice (Makin et al., 2022) and broader ERP guidance that power increases as trial counts approach ~90, with diminishing returns thereafter (Boudewyn et al., 2017; Clayson et al., 2019; Luck, 2014). A fixed-cohort subsampling procedure that equalized trial counts across participants showed that increasing the per-condition cap primarily narrowed confidence intervals without altering SPN morphology or mean amplitude—i.e., detectability scaled with precision rather than reflecting a change in the underlying waveform. Together, these observations address concerns about trial sufficiency and analytic generality: the sustained posterior effect replicates under a standard ROI/time-window definition, is robust to analytic approach, and remains stable in form while becoming more precisely estimated as trials accrue.

Finally, correlations between individual SPN magnitude and behavioral indices were weak and statistically inconclusive. SPN amplitude did not meaningfully covary with sensitivity or with decision criterion; frequentist and Bayesian summaries converged on no strong association. This invites caution rather than strong claims about absence of effect, as moderate relationships would require larger samples. A principled dissociation is plausible: the SPN appears to index the availability of structured perceptual information, whereas overt accuracy depends on downstream processes—including decision formation, confidence calibration, motor preparation, and fluctuations of attention—that need not covary tightly with mean SPN amplitude (O’Connell et al., 2012; Clayson et al., 2019; Dering et al., 2024). Individual variability may also reside more in timing or trial-level reliability than in mean amplitude, features that conventional averaging can obscure.

In sum, a clear SPN after a 20 ms presentation indicates that the visual system can initiate global-form integration with considerable temporal efficiency. A conservative interpretation is that an initial feedforward sweep rapidly flags structured regularities, after which recurrent interactions consolidate a coherent global representation (Palmer and Hemenway, 1978; Alp et al., 2018; Kohler et al., 2016). Our data support this two-stage account; they do not, however, adjudicate between specific pathway-level contributions, and we therefore avoid assigning the effect to particular channels without targeted manipulations (e.g., spatial frequency or chromaticity). Likewise, to minimize advance allocation of a fixed symmetry template, we randomized the relationship between the reference frame and the symmetry axis across trials; this design choice constrains top-down predictability without being central to the main inference about temporal sufficiency.

Lateralization was not a prominent feature of the present SPN. Although some studies report a right-hemisphere bias for symmetry-related neural dynamics, our sustained negativity was bilaterally distributed over occipito-parietal regions (Wright et al., 2015; Makin et al., 2021). This pattern fits with the idea that early, automatic stages of global-form processing are bilateral, with right-lateralized advantages emerging under sustained viewing or higher attentional demands. Neurostimulation studies are consistent with this graded view: perturbation of right lateral occipital cortex impairs symmetry detection more than left-sided stimulation but does not abolish it (Bona et al., 2014).

Two design features deserve brief comment. First, ERPs were computed from correct trials only, ensuring that the SPN reflects symmetry perception and addressing potential confounds from response bias (Bertamini et al., 2019). Second, the randomization of symmetry-axis alignment reduced the salience of a fixed template and limited pre-allocation of attention to a single axis. The persistence of the SPN under these circumstances supports accounts in which automatic symmetry processing operates in parallel with—and can precede—top-down enhancement (Machilsen et al., 2009; Treder, 2010; van der Helm and Treder, 2009).

Several limitations point to clear next steps. The exposure duration was not parametrically manipulated, preventing a direct characterization of the temporal growth of the SPN across brief-to-long presentations. Moreover, we did not employ backward masking; residual post-stimulus activity could therefore have supported the recurrent processes indexed by the SPN. Future work should combine graded durations with masking to delimit the effective processing window and to separate feedforward from feedback contributions to SPN generation. A further limitation concerns possible strategic focusing on a single symmetry axis under brief viewing: although elongation and oblique orientation were used to reduce fixed-template allocation, we cannot determine whether individual participants preferentially sampled only one axis (45° or 135°) on a given trial. Future studies should manipulate axis predictability directly, or include trial-wise reports of the attended axis. In addition, the practice dose was relatively large (80 trials versus 120 experimental) and was intended to stabilize task set and EEG signal quality; we did not quantify transfer from practice to the main task, so an influence of practice on sensitivity, criterion, or SPN cannot be ruled out. Such designs would also enable stronger inferences about channel contributions by varying spatial frequency content and chromaticity. Finally, larger samples will be needed to test for moderate brain–behavior couplings with adequate precision.

In sum, the present results show that the human visual system can register and sustain a neural representation of symmetry after a single, very brief exposure. A data-driven analysis isolated a posterior, sustained negativity overlapping the canonical SPN time course and topography, with no early differences and no significant anterior positivity. A conventional posterior ROI provided a conceptual replication within a reliability framework, and trial-count analyses demonstrated that additional epochs enhance precision without altering SPN amplitude or morphology. Comparisons with the SPN catalogue situate the effect within lower end of the expected bounds for task-relevant symmetry processing (Makin et al., 2022). Weak and inconclusive brain–behavior correlations further emphasize the distinction between perceptual availability and decisional performance.

## Conclusion

The current findings advance our understanding of the neural dynamics of symmetry perception. Even under extreme temporal constraints, the human visual system is capable of initiating and sustaining a perceptual representation of symmetry, as indexed by the SPN. This supports a two-stage model in which an early, automatic process flags global regularity, followed by later, recurrent stages that govern consolidation and decision-related operations. The decoupling of SPN amplitude and behavioral accuracy further highlights the importance of distinguishing between perceptual and decisional components in interpreting neurophysiological data. Taken together, the results underscore the efficiency and resilience of symmetry detection mechanisms and demonstrate that carefully optimized ERP designs can detect subtle neural effects even when the stimulus is presented for only 20 ms.

## Data Availability

The datasets presented in this study can be found in online repositories. The names of the repository/repositories and accession number(s) can be found at OSF, 10.17605/OSF.IO/BQGSC.
